# The importance of a good therapeutic alliance in promoting exercise motivation in a group of older Norwegians in the subacute phase of hip fracture; a qualitative study

**DOI:** 10.1186/s12877-020-01518-7

**Published:** 2020-03-30

**Authors:** Irene Vestøl, Jonas Debesay, Zada Pajalic, Astrid Bergland

**Affiliations:** Faculty of Health Sciences, Oslo Metropolitan University, Pilestredet, P.O. Box 4 St. Olavs Plass, 0130 Oslo, Norway

**Keywords:** Hip fractures, Exercise, Older people, Physiotherapy, Relationship, Therapeutic alliance, Participation, Motivation

## Abstract

**Background:**

Hip fractures represent a global public health issue that demands high cost both from the patient and from the society. Functional exercise in the subacute phase of a hip fracture is essential in reducing these costs. To the best of our knowledge, no qualitative study has explored the patients’ experiences in participating in an exercise program during the first month after surgery. Thus, this study aims to explore how older people who had participated in an evidence-based exercise intervention describe their relationship with their therapists and how this relationship might contribute to their motivation for exercise.

**Methods:**

Thirteen women and six men, who all had experienced a hip fracture and were staying in the same short-term rehabilitation unit, were interviewed by the last author. The interviews lasted from 30 to 70 min. The participants’ mean age was 86 years and they had all participated in a High Intensity Functional Exercise (HIFE) program in one-on-one sessions for 2 weeks, a total of 10 sessions. The recruitment was done by therapists involved in an RCT evaluating the HIFE-program with the attempt to obtain maximum variation. Data were analyzed through systematic text condensation in collaboration between all authors.

**Results:**

The analysis yielded three main themes integrated in the core theme “Therapeutic alliance is an interpretative filter for the participants’ experiences.” The three themes were “The feeling of mutuality and respect in the alliance”; “A trusting and motivating relationship” and “Tailoring of the instruction and program to make the task understandable”. These themes concerned basic needs in the relationship between the participants and the therapists which brought forward a feeling of mutual respect. The most prominent finding was the experience of trust in the therapists’ abilities, and how this contributed to the participants’ motivation to fulfil the program and achieve meaningful changes.

**Conclusion:**

Our findings suggest that therapeutic alliance is an indispensable aspect of a therapy, and relational knowledge and competence are prerequisites in the transfer of professional knowledge in a therapy. Our findings can be useful to therapists involved in clinical practice, especially to those working with vulnerable groups.

## Background

Hip fractures are a global public health issue that demand considerable human and financial resources. By 2050, the number of hip fracture cases worldwide will range from 7.3 million to 21.3 million annually [1]. In Norway, the number of hip fracture cases is approximately 9000 annually; the cost for a two-year treatment of one hip fracture ranges from 800,000 Nkr to 1,000,000 Nkr, and the mean hospital stay of a patient with hip fracture is four to five days. After hospital discharge, some patients are transferred for a short-term care in a primary health care unit for two to four weeks [2] before they can receive help at home. The important components of rehabilitation for hip fractures include functional training and physical activity, along with assessments to address depression [3] and undernutrition [4]. Individuals who remain sedentary after hip fracture are at risk for a new fracture and for further functional decline [5].

To a frail, older person, a hip fracture marks a decrease in quality of life, a poor outlook in life, and an increase in one-year mortality rate by approximately 30% [6]. A systematic review has estimated that 42% of survivors do not return to their pre-fracture condition, 35% become permanently incapable of walking independently [7], and 50% are expected to require assistance in their daily activities [8].

To achieve better survival rate, reduced pain, higher quality of life, and improved physical functioning, a functional exercise program should be initiated during the patients’ hospital stay and must be intensified when they are transferred for a short-term stay in a primary health care unit [9–11]. Tsauo et al. [12] have stated that there is a pressing need to understand and improve the process of functional recovery after hip fractures [13]. Today, hospital stays are short. Thus, subacute care soon after discharge plays an important role in promoting recovery. According to a systematic review and two Cochrane reviews [9, 14, 15], there are no set guidelines for best practice exercise programs after hospital discharge. Another systematic review has shown that structured exercises initiated a few months after fracture produced small improvements in overall mobility [16]. The efficacy of early-phase interventions after a hip fracture is much less studied compared with prolonged intervention after hip fractures. Hence, there is a knowledge gap on the effect and on the conduct of exercises in the sub-acute phase, that is, when the patients are in short-term care units following hospital discharge.

Hagsten et al. [17] have found that many older people see a hip fracture as the end of independent living. Therefore, psychological support from therapists during an exercise session may be necessary to encourage the patients and to show them that it is possible and harmless to perform ADL shortly after surgery. Since the patients’ ability to perform ADL/IADL is limited largely by psychological factors [18], patients’ education and motivation are vital concerns.

In clinical practice, the use of psychological techniques by physiotherapists has been suggested to be beneficial [19] in increasing their sense of self-efficacy and in promoting self-management among their patients [20]. Moreover, physiotherapists should consider employing a well-directed communication; they should also promote their patient’s self-control over their problem as well as avoid inadvertently reinforcing their patient’s pain behavior [20]. Studies in different physiotherapy fields have demonstrated that therapeutic alliance promotes positive treatment results [21–23]. In addition, a review on therapeutic alliance has verified that the role of physiotherapists is to activate the patient’s resources to manage the condition being treated [24]. These works [19–24] show the importance of the relationship between the physiotherapist and the patient, also called therapeutic alliance, in clinical practice. Theories on therapeutic alliance will further frame the present findings on an exercise intervention for older people in the subacute phase of hip fracture.

### Theoretical framework

Therapeutic alliance refers to the strong collaborative relationship between a person receiving therapy and another person providing it [25]. Edward Bordin [26] was one of the first scholars to formally describe therapeutic alliance; he noted that therapeutic alliance consists of three components: 1) the relationship bond between the therapist and the client, 2) the agreement on the goals of therapy, and 3) the agreement on the tasks of therapy. Borrowing from Bordin [26], other researchers have articulated the comprehensive role of therapeutic alliance, noting that it is an “inseparable part of everything that happens in therapy” [27]. Bordin’s framework [26] includes four propositions: 1) All genres of psychotherapy have embedded working alliances and can be differentiated most meaningfully in terms of the kind of working alliance each genre requires. 2) The effectiveness of a therapy is a function in part, if not entirely, of the strength of the working alliance. 3) Different approaches to psychotherapy are marked by the difference in their demands on the patient and on the therapist. 4) The strength of the working alliance is a function of the closeness of fit between the demands of a particular kind of working alliance and the personal characteristics of the patient and of the therapist.

Bordin [26] further proposed that the working alliance is one of the keys or *the* key to the overall process of change.

Researchers have investigated therapeutic alliance in clinical studies because of the consistent results on the association between an alliance and therapeutic outcomes [27–30].

Moreover, qualitative evidence demonstrates that positive relationships can facilitate engagement and can foster recovery [31]; consistent indications show that a therapeutic alliance is an important predictor of treatment retention, and it improves short- and long-term outcomes in patients with severe mental illnesses [32].

Therapeutic alliance in physical therapy has also been systematically investigated [33–35], and Taccolini et al. [33] focused on the physiotherapist–patient alliance. This review included four studies and found no strong relationship between therapeutic alliance and pain relief, but it highlighted the need for further studies on this topic. Hall et al. [34] analyzed 13 studies and found a positive correlation between the alliance and the treatment outcomes for pain, disability, physical and mental health, and satisfaction with the treatment. O’Keefe et al. [35] analyzed 13 qualitative studies and found that a mix of organizational, clinical, and interpersonal factors influences the relationship and interactions between a patient and a therapist. They also suggested that increased awareness of these factors by physiotherapists could enhance treatment outcomes. Given that therapeutic alliance constitutes a fundamental factor in effective therapy and that studies on physiotherapy are limited, there is a need to explore ways to facilitate therapeutic alliance, which is consistently related to therapeutic outcomes.

We found no studies investigating the therapeutic alliance between physiotherapists and older patients who suffer from hip fracture.

To the best of our knowledge, no qualitative study has explored the patients’ experiences in participating in an exercise program within the first month after surgery. Thus, this study aims to explore how older people who had participated in an evidence-based exercise intervention describe their relationship with their therapists and how such a relationship might have contributed to their motivation to execute their exercise regime. Due to the importance of this relationship in treatment adherence and in treatment outcome as shown in other therapeutic disciplines and patient groups [22, 27, 34–36], it is likely that such a relationship also is important when tailoring interventions for our patient group. Thus, this study potentially contributes valuable knowledge on how to encourage patient participation in an exercise regimen.

## Methods

We conducted a qualitative approach using semi-structured interviews to explore our patients’ experiences during the exercise sessions [37]. We wanted to get a grasp of the participants’ experiences from their lifeworld as they originally experienced them and to place our research in the phenomenological–hermeneutic tradition [38]. According to Bottorff [39], a qualitative inquiry offers unique advantages that contribute to the exploration of the complex process of research translation. Thus, we used the Consolidated Criteria for Reporting Qualitative Research (COREQ) checklist to ensure transparency in this study [40].

In Norway, some patients with hip fractures are transferred for a short-term stay in a primary health care unit [2] after being discharged from the hospital. These stays can be either a relief stay for the family/spouse or rehabilitative; a relief stay is free of charge [41]. Municipalities maintain these short care homes in different ways, but most of them employ municipality-paid physiotherapists who facilitate the patients’ rehabilitation process [42].

All the participants in this study stayed in a short-time rehabilitation unit for two weeks prior to returning to their respective homes after the hip fracture. Within this period, they all took part in the current exercise program.

### Ethical considerations

The Norwegian Regional Ethics Committee (2015/1814 REK sør-øst B) had given its approval for this study. The trial was registered in Clinical Trials Identifier: NCT02815254.

All of the 19 participants received written and verbal information about the large randomized controlled trial and this qualitative study, which received the same ethical approval. The participants were also informed about the possibility to withdraw their consent to participate at any point of the study, without any treatment consequences. All of the participants provided informed consent, and none of them withdrew their consent at any point during the study.

### Evidence-based exercise program

The exercise intervention our participants received was based on the High Intensity Functional Exercise (HIFE) program [43, 44], which became tailored to the individual participant. Each exercise session included a five-minute seated warm up, at least two exercises that strengthen the lower limb, and two balance exercises. The HIFE exercise program consists of a selection of functional exercises divided into the following categories: 1) dynamic balance training combined with strength training for lower limb, 2) dynamic balance training while walking, 3) static and dynamic balance training exercises while standing, 4) lower limb strengthening exercises with continuous support, and 5) gait training with continuous support [43]. The therapists encouraged high intensity and progression, and there was an opportunity for this in each exercise by using a weighted belt. This was tailored to the individuals’ functional level and current health situation. Four physiotherapists were involved in this intervention, and they served as instructors to our participants. Two out of the four therapists were females (38 and 40 years old) and had received continuing education in geriatrics. The female therapists had 12 and 13 years of experience in geriatric rehabilitation therapy. The male instructors were 30 and 39 years old and had respectively six- and 12 years’ experience in geriatric rehabilitation.

### Participants

This qualitative study followed an RCT nr.: NCT02815254 where the aim was to evaluate the HIFE program in Eastern Norway. Nineteen older people with mean age of 86 years, all in the same unit were recruited through purposive sampling, with an attempt to observe the maximum variation in characteristics including age, sex and functionality. (Table 1, additional file 1). The physiotherapist at the unit engaged the participants consecutively in parallel with the trial and the inclusion criteria were the same as for the clinical trial as follows: aged 65 years and older, living at home, able to walk independently indoors with or without an assistive device, and able to provide informed consent to participate in this study. The exclusion criteria were dizziness that affect their participation in the exercise program. To decide the number of participants, we were guided by Malterud’s model of information power, which depends on the specific study aim, sample specificity, use of established theory, dialogue quality, as well as the strategy of analysis [45]. Malterud [45] further suggests 10–25 participants to be sufficient sample size in qualitative studies. After completing 18–19 interviews, no substantially new information was obtained, and the study reached saturation.

### Procedure of recruitment and interviews

The physiotherapists performing the intervention in the RCT provided information about this qualitative study as well, and invited patient to participate when they came to the unit. When consent to participate was given, the interview was scheduled by the last author consecutively. Data were collected from October 2017 to Mai 2018 and all the patients who were asked within this time period, agreed to participate. The interviews were conducted on the last day of their stay in a primary health care unit where they had received daily one-on-one exercise sessons for 10 days. The interviews lasted for 30–70 min and were conducted in the patients’ rooms at the care home, with only the interviewer and the participant present. The participants knew that the interviewer was a physiotherapist and an experienced researcher and we had prepared a semi-structured interview guide, with questions like: “Could you please tell me about your experiences of participating in the exercise program after the hip fracture?”, “Could you please tell me about the instructor/physiotherapist?” and other questions available in the interview-guide (additional file 2). The interviewer wrote notes during the interviews as a tool to formulate follow-up questions.

### Measurements

To measure the functional characteristics and health-related quality of life of the participants, we used the following assessments: To assess function, we used the Short Physical Performance Battery (SPPB) assessment tool, which includes three subtests (balance, walking speed, and repeated chair sit-to-stand test) that were scored based on scale of zero to four–; the total score ranges from zero to 12, and high scores reflect better function [46]. Cognitive function was evaluated using the Mini Mental State Examination (MMSE), scored based on a scale of zero to 30 [47]. Health-related quality of life was assessed using the Short Form-12 (SF-12). The SF-12 physical and mental health summary measures are referred to as PCS-12 and MCS-12, respectively. The SF-12 data were analyzed according to the instruction manual and based on the sum score for PCS-12 and MCS-12 [48]. In all of the assessments used, a higher score corresponds to better functioning. The characteristics are listed in Table 1 (additional file 1).

### Analysis

All interviews were audiotaped and transcribed in Norwegian by an external professional transcriber; the transcript consisted of a total of 348 pages. All authors read through the raw data to get a total impression of what it could tell about the aim of the study. In this process, parts of the data were discarded for analytic reduction [45]. Furthermore, the first author coded the data concerning the relationship between the therapists and the participants to search for patterns of commonalities and differences in the participants’ experiences. Translations from Norwegian to English was done in the end of the analyzing process by the first author in collaboration with the co-authors. The analysis followed Giorgi’s phenomenological method [49] as modified by Malterud [45]. This process started with the authors engaging in a naive reading of the transcripts. The next process was the capturing of key concepts and thoughts; to do this, the text was read thoroughly to extract the meaning units, focusing on the patients’ experience of their relationship with their therapist during the implementation of the exercise program. The meaning units were coded and grouped into categories by using a coding scheme. All steps of the analysis were discussed and agreed upon by all authors. Finally, from the analysis, the descriptions fell into three main categories. At this stage, we framed an overarching theme, that is; “Therapeutic alliance is an interpretative filter for the participants’ experiences”; this theme summarizes the most illuminating findings from the data. This form of analysis is theoretically anchored in the phenomenology and is based on the participants’ lifeworld as they experience it [45]. In the end, three themes emerged from the analysis, namely: “The feeling of mutuality and respect in the alliance”, “a trusting and motivating relationship” and “tailoring instruction and program to make the task understandable”. The steps in the analysis process are shown in Table 2 (additional file 3). To ensure the rigor of the analysis, all of the authors have agreed upon the final version of the analysis and of the categories.

Trustworthiness and the evaluation of the worth of data in qualitative research implies establishing credibility, transferability, dependability and confirmability [50]. To achieve credibility in this study, the quality of the interviews and analyses should be examined. All authors were actively involved in both analysis and interpretation and strove to become aware of our preunderstandings and our influence on the emerging findings, as well as in how well the categories represented the data. The transcribed material seemed to generate thick descriptions, addressing the research aims. The selection of participants, the data collection, and the data analysis were executed as carefully as we managed, to enhance credibility of the study. The extent to which the findings can be transferred to other contexts, settings or groups [50], was achieved by providing an in-depth, detailed, and descriptive analysis of the data and by quoting the participants’ responses to substantiate the findings, which according to Lincoln and Guba can provide this type of external validity [50]. Dependability concerns whether the findings are consistent and stable [38]. One researcher conducted all the data collection in this study. Therefore, there is consistency between and throughout the empirical data in study. Lincoln and Guba stress the close ties between credibility and dependability, arguing that, in practice, a demonstration of the former goes some distance in ensuring the latter [50]. Confirmability refers to the degree of neutrality or the extent to which the findings are shaped by the respondents and not by the researchers’ bias, motivation or interest. To ensure concordance between the content of the interviews, we have illustrated the themes using quotations assigned with a code that refers to the person who made the statements. The first author, a female physiotherapist, was also aware of the physiotherapist lens she possesses.

## Results

Our participants were 13 women and six men with age ranging from 72 to 95. Twelve out of 19 were living alone at the time, while five used rollators as a walking aid and 14 used a pulpit aid. The MMSE score ranged from 16 to 29 where 30 is maximum. The mean MMSE-score for the participants was 23. The SPPB scores ranges from zero (worst performance) to 12 (best performance). Our participants scored from one to nine on this test. The mean score was 4.7. A score lower than 10 indicates one or more mobility limitations. For more details about characteristics, see Table 1 (additional file 1).

In the following section we will present the result of the analysis of the interviews.

Our analysis yielded three main themes that were integrated into the overarching theme of this work “Therapeutic alliance is an interpretative filter for the participants’ experiences”. The therapeutic alliance emerged as an interpretative filter for the participants’ experiences regarding their trust in their therapists’ competences and in their own abilities to achieve positive changes and accounts for the most prominent finding of this paper. Being an interpretative filter, alliance highlights the importance of different aspects of the relationship between the participants and the therapist, which promote the participants’ sense of control over their problem, the agreement regarding the goals of therapy, and the task of therapy; moreover, the alliance was important in the patients’ attempt to cope with their situation during recovery. The patients’ situations were mostly seen as stressful and exhausting; however, the relationship of the patients with their therapists was rewarding and kept them going. The three main themes, along with the selected quotations from the interviews to illustrate the findings, are presented below.

### The feeling of mutuality and respect in the alliance

The therapists’ ways of being were described in terms of their actions and their personal characteristics and how these features helped them address the participants’ needs for explanations and support in their current situation. The one-on-one sessions implemented in this exercise program created the feeling of being in a collaborating team, which was described by several participants, as such in the following:

“I felt that the physiotherapist and I are a team or an alliance. To be a bit pompous, we are a team. I have respect for the therapists, and I have learned that they respect me. I don’t get infected by hopelessness” (male, 90-99).

The relationship between the therapist and the patient is further described as an equal and mutual interaction, although they have different backgrounds and power in the situation. This mutuality was described in connection to respect, effort, and goals. Examine the following quotation on the commonality of goals:

“Our common objective is for me to be able to come home again; at the same time, my personal objective is to be able to walk better” (male, 90-99).

Efforts from both sides — the physiotherapist and the participant — were needed “to make magic happen” as described below:

“I feel that I am listened to in the face-to-face session. We talk about my situation in relation to the exercises. My effort is the most important in this endeavor, but without the therapist’s contribution, nothing would have happened. It’s the common effort that makes it happen” (male, 90-99).

The participants were all in a vulnerable situation and needed support, comfort, and help to mobilize their own will to implement changes in their lives in order to find motivation for exercise:

“She treats me with respect. I believe in her firmness—she never forces me, but she makes me give my all. She managed to mobilize my will. I know that this was good, even if it is exhausting” (female, 70-79).

Many of the participants also talked about the feeling of being “in charge” in the team regarding the decisions concerning their own lives, though the therapists were firm in facilitating the exercises. The feeling of being in a team or co-ownership, as one participant put it, contributed to their motivation by empowering them to exercise control over their lives, as indicated in the following:

“To feel this co-ownership is important to me. I like to be a part of it and to be taken seriously. The therapists in this project do so. They possess much knowledge on people, communication, motivation, and information and on how to listen to what I say. That is important. In my experience, they don’t just listen; they take my words into account” (female, 70-79).

The creation of a good relationship in the team or in the alliance helps in the translation of the instruction into useful information as described in the following:

“The exercise is a mutual project. It’s a very helpful exercise towards my mastery of everyday life” (male, 90-99).

### A trusting and motivating relationship

A good relationship based on trust was formed between the participants and the therapist as richly described in the data. Apparently, this good relation is important in the instruction and implementation of the exercise program, as indicated in the following:

“We have achieved a good relationship, the physiotherapist and I” (male, 90-99).

The participants’ experiences of this relationship describe an atmosphere of trust, acceptance and being seen, which apparently have motivated them to complete the program.

The participants talked about trust in connection to human qualities like respect and acceptance, which they experienced that the therapists showed them. Altogether, these human capabilities gave the participants confidence in themselves and strength to carry on with the exercises. The physiotherapists’ ability to turn negative attitudes into positive ones by providing trust was evident in the participants’ words, as in the following:

“Yes, I have developed trust in the exercise. I know that I was not so motivated when I started out. I was scared, and I realized that being old is demanding in itself, and then, with the hip fracture along with the tiredness, I was downhearted and not sparkling and optimistic. But the therapist accepted it and did not “drop” me; I became less depressed or became more optimistic after these meetings” (female, 80-89).

Further, the concept of trust was visible in the participants’ words according to intensity and progression. The therapists were concerned about how the participants progressed in the exercises in the context of a mutual process, that is, by identifying and describing even a small progress, they tend to increase the participants’ motivation. Given the differences in the participants’ starting points, in their level of pain, and in other characteristics as described earlier, the progress was timed and tailored to fit each participant. Moreover, the therapists were responsive to how much pushing each individual could take, as in this description:

“I know my therapist is interested in the intensity of the exercise and wants to increase the intensity if possible. Intensity is connected to how much I can take. I trust the therapist that it will not be dangerous for me. I like making progress, especially when I experience it, but I come to a point when I must strain a little more … I have managed more repetitions and heavier weights. To walk up the stairs, get rid of the walking aids” (male, 90-99).

The therapist’s knowledge of hip fracture and the patients’ post-operative situation gave the participants the opportunity to experience professional treatment as well as relational understanding and acceptance. The following quotation implies that the therapist also demonstrated psychological skills.

“I feel that the therapist understands my situation with my hip fracture. I feel accepted, and she sees me as a person. She shows compassion and gives the help we need. Being with her puts me into a good mood. One could imagine that she is a psychologist” (female, 80-89).

With respect to positive and caring pushes, the participants described their therapists’ ways of motivating and giving encouragements as suitable for their vulnerability*.* In addition to feeling down psychologically, most of the participants experienced physical pain from their fracture during the exercise, making it even more difficult for them to put the effort needed for the exercise. The therapist’s manner of explaining the pain mechanisms and the significance of each exercise motivated the participants to execute the exercise in spite of their pain. Thus, encouragement and positive feedback meant a lot to the participants, such as in the following:

“My therapist is clever in motivating me. It is difficult to be motivated when you are in pain. You need these small positive pushes to recognize the benefit of the exercise and to realize that it can give you results. She focuses on what is important in the exercise, talks as much as needed, and sees me as a person if I have a bad day. She makes note of that, but still she gives me the benefit of the doubt. A hip fracture makes you feel really bad sometimes, especially in the acute phase. I am happy for this opportunity and the support from the therapist who helps me see the light at the end of the tunnel” (male, 70-79).

The participants described the physiotherapists as likable, nice, and mild persons who were always in good mood and who created a happy atmosphere during the one-on-one sessions. They were also described as calm, loving, and empathic. Their ability to show interest to and involvement with them during the exercise session meant a lot, promoted positive emotions, and helped them to execute the exercises, as evident in the following quotation:

“The fact that I liked my therapist’s attitude meant a lot. The stress level gets lower as a result; I feel that she cares about me, and I can put my trust in the program based on her explanations. She seems empathic and is concerned about my condition. She makes my mind turn away from myself to what I’m doing” (male, 90-99).

To be treated with an understanding attitude seemed very important considering the participants’ vulnerability. The participants appreciated the physiotherapists’ helpfulness, patience, and display of interest and understanding, and these qualities were described to promote motivation, as a participant puts it:

“I have felt that I get attention and that they cheer me up. But it has its costs, indeed” (male, 90-99).

Another participant said that the caring manner by which her physiotherapist presented the exercise program took away her resistance toward the activity, and it helped her to receive the knowledge shared by her physiotherapist and to realize how important the exercise was for her:

“The way they talk about the exercise makes me lose my resistance toward it. They make me realize that the exercise is important to me. I do the effort for myself, not for them. They see me, and they show that they do. They don’t talk over my head but with me, and they are interested in what I think and in how I am doing” (female, 70-79).

### Tailoring of the instruction and program to make the task understandable

The tailoring of the program both in terms of verbal and practical instruction was appreciated by the participants. In their words, this tailoring provided understanding and helped them execute the program. Tailoring of the verbal instruction facilitated understanding of the exercises and of the program as a whole.

The participants gave positive feedback on the therapists’ presentation and guidance in the program implementation, particularly in their firmness, in their provision of credible information and explanations, and in their being good listeners, as in the following:

“She is good at giving information and answering my questions “(female, 70-79).

A 90-year-old female participant described how the life of an old person can be hard enough in the first place and how an older person didn’t have too much extra capacity to do things. She felt that the therapist tuned into her mood and used it as a basis in facilitating the exercise. The majority of the participants expressed how the therapists individualized or tailored the lessons to help them understand the significance of each session. They were described as good communicators with pedagogical skills. A male participant said the following:

“She does not play expert but presents the message on a level that makes it suitable for me” (male, 80-89).

It was also underlined that the therapists entertained all kinds of questions. No questions were too stupid. Their being good listeners and trusting also contributed to the patients’ motivation. Moreover, the therapists made sure that their message was understood and gave the right information at the right time during the instruction sessions, as evident in the following statements:

“They do active listening, and they explore what I think and whether I have understood the information they have provided. They talk simply and do not use too many difficult words” (female, 80-89).

“She is clever to give easy explanations initially and then provides more information as necessary” (male, 80-89).

“The therapist is good in motivating me; she tells me how things can be done more easily. She gives me praises and tells me what I have achieved. She challenges me and talks to me in an understandable way, which is important” (male, 90-99).

The tailoring of the specific movements in the exercises went hand-in-hand with the tailored instruction and was also described widely in the transcript. They felt as though their therapist had designed or redesigned the program to fit each individual’s needs in terms of intensity, goals, and progress. This tailoring was important for the participants, as expressed in the following:

“My motivation is attributed to the being open of the therapists to my needs, even though the program is standardized in a sense. They allowed it to be tailored to my needs, so it became relevant and perfect for me” (male, 90-99).

The participants’ important roles and life areas mentioned in the data were put into the context of physical activity, and the physiotherapists expressed support and explained how the exercises could help them achieve better functioning to fulfil these roles, which were important in their lives. This is evident in the words of one participant, as follows:

“They put the exercise into the context of what I need and like to do in my daily life. The exercise seems suitable to help us to get dressed, to wash ourselves, to stand up from a chair and last but not least, to walk” (female, 80-89).

The support received from the physiotherapists was also evident in the participants’ description of the significance of the instruction and of the dialogue during the exercise to their own lives. Apparently, during their dialogue with the participants, the physiotherapists actively used the participants’ personal objectives to motivate the latter to execute the exercise, as expressed in the following quotation:

“I realized that she has useful knowledge that can contribute to my being able to continue to work in the garden and out on my brother’s farm. She gives me faith to believe that I can make progress and that it never is too late” (male, 80-89).

The creativity of the therapists in finding alternative ways to facilitate specific movements when the participant struggled to execute them the original way was evident in the data, as follows:

“The physiotherapists managed to tailor the program. They know a lot about the body—how to get it started again — and they have a lot of smart tricks; if I couldn’t manage to get up from the chair, they put a pillow under my rear end, and then I felt what it was like to stand up. Since I didn’t have any contact with my muscles—the pain paralyses you—the tailoring process is important” (female, 70-79).

## Discussion

To the best of our knowledge, this study is the first to investigate how older people in the subacute phase of a hip fracture describe their relationship with their physiotherapists and how this relationship has contributed to their motivation to exercise.

The overall results highlight the importance of achieving a good therapeutic alliance. The participants noted that certain features of the alliance, such as mutuality and respect as well as trust, aspects related to balance of power in the relation and understandable communication, were especially important in establishing the proper instruction of exercises in the early phase of recovery after suffering a hip fracture. A relationship between a physiotherapist and a participant based on mutuality, respect and trust, wherein the therapist acts as the agent of change and the participant is the one who is seeking change, can be described as a therapeutic alliance [26]. We found Bordin’s [26] theory on therapeutic alliance suitable for framing our results, given that a therapeutic session is an avenue for change. Studies in physiotherapy have found that a therapeutic alliance can potentially improve the effects of physiotherapy [22, 24, 34–36, 51, 52], however, they underline a lack of description of this alliance as well as how it could be improved [22, 34, 36]. Therefore, our study can contribute to the literature via rich and detailed descriptions of how the physiotherapists can make patients feel like being part of a team and how their instruction styles can encourage an atmosphere of respect, trust and understanding.

Three important components of a good; professional relationship were detailed by Røkenes and Hansen [53], including way of being, empathy, and recognition [53], which are easily relatable to the mutuality and respect that were described by our participants. These features are also important existentially and thus must be considered when building a professional relationship to patients. The authors stated: “We are all vulnerable, and professionals’ ways of being can hurt the self” [53] (page 179).

Our results are supported by those of Hubble, Duncan, and Miller [30], who characterized the relationship of a therapeutic alliance as when clients feel heard, understood, and respected; moreover, they defined the goal component of a therapeutic alliance as targeting what the client wants to address in treatment [30]. It seems that the respectful attitude that our participants experienced from their therapists was a prerequisite to their feelings of mutuality, such as when they expressed that they felt their sessions were like “being on a team” or “in an alliance” with the therapist or having “co-ownership” of the physiotherapy process. The participants felt that their words were heard and considered by the therapists, and they were given the “chief-position” in decisions concerning the exercise. The therapists managed to reflect the participants’ own needs and desires in a respectful manner as they gave exercise instructions. In our case, the alliance became is interwoven and an integral component of a successful therapy. Our results align with Bordin’s [26] aspects of goals, tasks, and bonds as well as with the idea that the outcome of a therapy is more or less a function of the strength of the alliance and the closeness of fit between the demands and the personal characteristics of the participant and the therapist [26].

Trust is an important element in the creation of bonds in a therapeutic alliance [26]. Our participants widely described the trust that they felt in both the therapists and the program. Other studies have also shown that trust is important in the working alliance from the perspectives of the therapists as well the patients [20, 54, 55]. Furthermore, Røkenes and Hansen [53] underlined the importance of their therapists’ ability to act as a container for the patients’ feelings, which is important in the development of a strong and trusting bond in the alliance; this concept was also pointed out in Bordin’s [26] conceptual framework. Trust is possibly more important for patient groups that are particularly vulnerable, as our participants, who described downheartedness, being alone, fear of falling again, and pain among other feelings of being put out of charge over their lives. This aligns with other research on establishing a therapeutic alliance in physiotherapy involving patients with chronic pain disorders [19, 22, 33, 36, 52]. In 2019, Calner et al. [56] interviewed persons with persistent musculoskeletal pain and found that the alliance between the physiotherapist and the patient must be built on initial trust, a continuous dialogue, and clear communication. This finding is consistent with that of O’Keefe et al. [35] and Slade et al. [57], who found that the alliance factor is an element that can enhance treatment outcomes. Harman et al. [20] found that trust is an important element in a therapeutic alliance from the physical therapist’s perspective, and point of view, and is related to the Motivational Interview’s principles of expressing empathy. Furthermore, they found that placing trust in their physiotherapist helped patients continue with their program, which was becoming more difficult during the therapy period and was not creating immediate results. The same phenomenon emerged from our data. For example, a 95-year-old male patient described his therapist’s intention to increase the intensity of the exercise, and he verbalized his trust in his therapist’s competence as well as that he believed that the increase did not represent any danger to him.

Another aspect that is important in the building of bonds is the power relationship [58]. The professional relationship between a therapist and a patient can be characterized as complementary, in which the two have different levels of power and roles [53]. In a complementary relationship, the imbalance of power is natural due to differing roles. While, the differences are known to and accepted by both parties, the professionals’ proper use of power significantly affects how the relationship will be experienced by their patients.

In a good and trusting relationship between a patient and a professional, the latter can allow himself/herself to be critical toward the patient without jeopardizing the relationship [53]. This was indicated in our participants’ statements of feeling challenged by the therapist’s firmness and that the exercise had its costs. They expressed trust in the therapist’s words and felt that their resistance to performing the exercises was eliminated by the therapists’ approach to the therapy. Interestingly, the participants felt that they were “in charge”, despite their vulnerability and the imbalance of power in the relationship. Holopainen [55], who investigated the encounters of patients with low back pain in the health care system, had a patient state the following to healthcare professionals: “Put us in charge, with you working as a coach” (page 275). Nevertheless, our participants were in fact vulnerable, and our findings suggest that their vulnerability made trust essential. They were older persons who had been hospitalized due to a hip fracture caused by a fall, and their injuries were not manageable at home. The incident represented a serious change in their lives. After being discharged from the hospital, our participants were transferred to a short-term care home, where they agreed to participate in the current intervention, which demanded both physical and psychological changes from them.

In Latin, communication means “to share” [53]. Sharing presupposes a relationship, which must occur in all kinds of communication. Further, respect and trust can be considered prerequisites of good communication. The communication that occurred in the alliance between the therapists and the participants in our study helped the latter find meaning in their exercises and understand and complete the program. This finding was supported by a meta-analysis [27] involving nearly 70 studies from the psychotherapy field. Røkenes and Hansen [53] underscored the significance of being good listeners, of being able to communicate clearly and of letting the participant come forward with his/her difficulties in a professional relationship. Hubble, Duncan and Miller [30] also described the relationship component of a therapeutic alliance as when clients feel heard and understood. The therapists in our study did succeed, according to these abilities, which the participants described as being seen and heard, with none of their questions considered stupid. All these abilities were mentioned by our participants and were of great importance in their completion of the program.

Del Re et al. [27] summarized that a therapeutic alliance is indispensable in all aspects of any therapy. We have tried to describe how a therapeutic alliance is experienced by older patients in physiotherapy and explained that our findings indicate that respect, trust and clear communication are essential factors in a therapeutic alliance that is capable of facilitating and helping older patients perform exercises that have been proven effective.

### Strengths and limitations

Our findings are context-bound to the participants and study setting, similar to all quality research [59]. This article is built upon a large data material that offers extensive descriptions of therapeutic alliance as a phenomenon. The participants’ testimonies were particularly rich in content given that data were obtained through face-to-face interviews by an experienced researcher. This represents a strength of this study’s credibility but can also be a limitation given the possibility that the participants may have given socially desirable responses which cannot be discounted. Though all researchers were as explicit about our preunderstanding and existing knowledge of the context as we could, our preconceptions may have influenced the research process regarding the credibility and confirmability of the study.

Our participants’ views concerns physiotherapists and may only to a limited extent be transferable to therapeutic alliances involving other health professionals. Particular patient groups, such as patients from ethnic minorities, patients with severe cognitive impairments, and aphasia, were not included in the sample. These factors may pose a limitation to the transferability of the study. Moreover, we conducted this study in one single rehabilitation unit placed in an urban area, which may have limited the transferability to other units in more rural areas accordingly. Although there exists a diversity between short-term rehabilitation units and various organizational models [60], they are comparable because they have the same mechanisms regarding purpose, structure, function and content [61]. We thus believe our findings will have great implications in similar short-term rehabilitation units.

### Implications for practice

The present findings may be useful to all physiotherapists involved in clinical practice, especially to those working with vulnerable groups and to leaders organizing health services. Moreover, emerging themes may be transferable to other clinical settings and populations and be useful to clinicians and students.

Our participants underline the importance of being respected as a person and being met in a one-on-one session to feel trust and motivation to execute the exercise program. This finding may have implications in the organization and prioritization of health care services, especially in the area of physiotherapy.

## Conclusion

Our findings underline the importance of a good therapeutic alliance involving professional skills, respect, trust, and acceptance in the achievement of positive outcome from a physical exercise treatment. These traits were operationalized in particular ways of communication and in tailoring the instructions and the program to fit an individual’s needs. Our participants’ overall situation may have increased their need for help to get motivated, which therapists have managed to address according to the patients. Furthermore, our findings suggest that therapeutic alliance is indispensable in all aspects of the therapy and that relational knowledge and competence are prerequisites in the transfer of professional knowledge in a therapy. Our participants felt they had a strong, positive alliance or relationship with their therapists, and this relationship has contributed to their motivation to complete the program and experience recovery.

## Supplementary information

**Additional file 1: Table S1.** Demographic and functional characteristics of the participants.

**Additional file 2.** Semi structured interview guide.

**Additional file 3: Table S2.** Examples of the analysis process.

## Data Availability

Data and material can be obtained by contacting the corresponding author.

## Abbreviations

ADL: Activities of Daily Living
IADL: Instrumental Activities of Daily Living
HIFE: High Intensity Functional Exercise program
SPPB: Short Physical Performance Battery
MMSE: Mini-Mental State Examination
SF-12: Short Form-12 test
PCS-12: Physical Component Summary (SF-12: The Short Form-12 test)
MCS-12: Mental Component Summary (SF-12: The Short Form-12 test)
REK: Norwegian Regional Ethics Committee
COREQ: Consolidated Criteria for Reporting Qualitative Research, a checklist
