# Supplementary material for: The importance of a good therapeutic alliance in promoting exercise motivation in a group of older Norwegians in the subacute phase of hip fracture; a qualitative study
Source: BMC Geriatr. 2020 Mar 30;20:118. doi: 10.1186/s12877-020-01518-7 (PMC7106840; doi:10.1186/s12877-020-01518-7)
Supplement: Supplementary file 1 — Additional file 1: Table S1. Demographic and functional characteristics of the participants. [file 12877_2020_1518_MOESM1_ESM.docx]

| Informant | Age-group | Gender^a^ | Living alone^b^ | Walking aid^c^ | MMSE^d^ | MCS12^e^ | PCS12^f^ | SPPB^g^ |  |
| --- | --- | --- | --- | --- | --- | --- | --- | --- | --- |
| 1 | 80-89 | F | N | 2 | 17 | 54 | 31 | 9 |  |
| 2 | 80-89 | F | Y | 2 | 26 | 37 | 36 | 6 |  |
| 3 | 90-99 | F | N | 1 | 24 | 53 | 41 | 2 |  |
| 4 | 70-79 | F | Y | 2 | 29 | 44 | 34 | 9 |  |
| 5 | 80-89 | M | Y | 2 | 22 | 35 | 30 | 5 |  |
| 6 | 80-89 | M | Y | 2 | 20 | 47 | 37 | 3 |  |
| 7 | 70-79 | F | N | 2 | 29 | 48 | 22 | 3 |  |
| 8 | 90-99 | F | Y | 2 | 23 | 51 | 25 | 1 |  |
| 9 | 90-99 | M | Y | 2 | 29 | 49 | 28 | 9 |  |
| 10 | 80-89 | F | Y | 2 | 26 | 37 | 35 | 6 |  |
| 11 | 90-99 | F | Y | 2 | 21 | 42 | 47 | 3 |  |
| 12 | 80-89 | F | Y | 1 | 29 | 50 | 38 | 6 |  |
| 13 | 80-89 | F | N | 1 | 17 | 31 | 54 | 9 |  |
| 14 | 80-89 | M | N | 2 | 16 | 48 | 52 | 3 |  |
| 15 | 80-89 | M | N | 2 | 25 | 39 | 36 | 1 |  |
| 16 | 90-99 | F | Y | 1 | 18 | 41 | 41 | 3 |  |
| 17 | 80-89 | F | Y | 2 | 24 | 48 | 38 | 5 |  |
| 18 | 90-99 | F | Y | 1 | 20 | 43 | 45 | 4 |  |
| 19 | 70-79 | M | N | 2 | 16 | 52 | 48 | 3 |  |
| ^a^ M=Male, F=Female, ^b^ Y–Yes, N–No , ^c^1–rollator, 2–pulpit aid; ^d^Mini Mental State Examination score; ^e^Health-related quality of life; ^f^meters per minute; ^g^Short Physical Performance Battery score | | | | | | | | | |

Table 1. Demographic and functional characteristics of the participants
