# Supplementary material for: The importance of a good therapeutic alliance in promoting exercise motivation in a group of older Norwegians in the subacute phase of hip fracture; a qualitative study
Source: BMC Geriatr. 2020 Mar 30;20:118. doi: 10.1186/s12877-020-01518-7 (PMC7106840; doi:10.1186/s12877-020-01518-7)
Supplement: Supplementary file 2 — Additional file 2. Semi structured interview guide. [file 12877_2020_1518_MOESM2_ESM.docx]

**Semi-Structured Interview Guide: Hip fracture, qualitative study**

Would you please tell me about your life situation now ?

Would you please tell me more about what fracturing your hip meant to you?

Could you please describe the fall situation?

How did you cope when you fell*?*

Would you please tell me about your life before you fell and fractured your hip.

Could you please tell me about your experiences of participating in the exercise program after the hip-fracture.

Could you please describe some of your reasons for participating in the exercise program?

Could you please tell me to what extent did you feel ready to engage in exercise following your hip fracture?

Could you please tell me what was most helpful about the exercise program?

Is there anything in particular that you remember from the exercise program that has been important to you?

Could you please tell me about your exercise instructor/physiotherapist?

If so, in what way did they motivate you?
Could you please tell me if you set goals for the exercises alone or together with the exercise instructor/physiotherapist?
Could you please tell me to what extent did the exercise instructor/physiotherapist direct you in your choices?
Did you meet your goals?

Could you please tell me what the exercise has meant to you?

Could you please tell me what you think about the future?

Could you please tell me how your activity level change after the exercise program had finished?
Could you please tell me if you kept motivated to do exercise?

If so how?

Could you please tell me if you were able to modify something about the exercise program, what would it be? What could be improved?

Could you please tell me, if you made any changes to your life in order to avoid falling another time?

If so what are these changes?

If you were to give advice to someone who had recently sustained a hip fracture for the first time such as you, what would that advice be?
Have other important people in your life been supportive in your recovery from the hip fracture?
Could you please tell me how important other people are in influencing your recovery?
What was helpful about that support?
