# Supplementary material for: The importance of a good therapeutic alliance in promoting exercise motivation in a group of older Norwegians in the subacute phase of hip fracture; a qualitative study
Source: BMC Geriatr. 2020 Mar 30;20:118. doi: 10.1186/s12877-020-01518-7 (PMC7106840; doi:10.1186/s12877-020-01518-7)
Supplement: Supplementary file 3 — Additional file 3: Table S2. Examples of the analysis process. [file 12877_2020_1518_MOESM3_ESM.docx]

| **Participant** | **Meaning units** | **Codes** | **Condensation** | **Theme** |
| --- | --- | --- | --- | --- |
| 9 | My effort is most important in this, but without the therapist’s contribution, nothing would have happened.  It’s common efforts that make it happen. | Own effort  Therapist’s contribution  Common effort | To realize the exercise, contribution from both the therapist and me is needed, though my effort is the most crucial.  Common effort makes it happen | The feeling of mutuality and respect in the alliance. |
| 7 | To feel this co-ownership is important to me.  I like to be a part of it and to be taken seriously. The therapists in this project do so.  They possess much knowledge on people, communication, motivation, and information and on how to listen to what I say. That is important. In my experience, they don’t just listen; they take my words into account. | Important co-ownership  To be taken seriously  People knowledge  Communication skills  Motivational skills  Listening skills  Taking words into account | Co-ownership with the physiotherapist is important.  I feel like I am taken seriously.  The therapists possessing important knowledge and listening to what I say. | The feeling of mutuality and respect in the alliance. |
| 7 | She treats me with respect. I believe in her firmness—she never forces me, but she makes me give my all. She manages to stir me to action. I know that this was good, even if it is exhausting. | Respectful treatment  Belief in firmness  No force  Mobilization of will.  Good but exhausting. | Her firmness makes me give my all, but she respects my boundaries. | A trusting and motivating relationship. |
| 19 | My therapist is clever in motivating me. It is difficult to be motivated when you are in pain.  You need these small positive pushes to recognize the benefit of the exercise and to realize that it can give you results. | Therapist is a good motivator.  Motivation is difficult when one is in pain.  Positive pushes.  Benefits of exercise. | My therapist motivates me in spite of my pain.  To be motivated, I need to see the benefits of exercise. | A trusting and motivating relationship. |
| 5 | She is clever in giving easy initial explanations  and then provides more information as necessary. | Cleverness in providing information. | The physiotherapist provides the right information at the right time. | Tailoring instruction and program to make the task understandable. |
| 9 | I know my therapist is interested in the intensity of the exercise and wants to increase the intensity if possible. | Intensity.  Progression. | Increasing the intensity of the program is important for my therapist. | Tailoring instruction and program to make the task understandable. |
| 9 | The therapist is good in motivating me; she tells me how things can be done more easily. She gives me praises and tells me what I have achieved. | Good motivator.  Good at telling how things can be done more easily.  Giving of praises. | The therapist motivates me by providing explanatory and encouraging instructions. | Tailoring instruction and program to make the task understandable. |
